# Genome-Wide Identification and Expression Analysis of the Aspartic Protease Gene Family and Their Responses to Abiotic Stress in *Talaromyces marneffei*

**DOI:** 10.3390/microorganisms14071477

**Published:** 2026-07-06

**Authors:** Santao Zhao, Jingliang Chen, Yeyang Zhang, Jingyi Ou, Youchao Dai, Feilong Xu, Pengle Guo, Xiaoping Tang, Linghua Li

**Affiliations:** 1 Infectious Disease Center, Guangzhou Eighth People’s Hospital, Guangzhou Medical University, Guangzhou 510440, China; zhaost095@126.com (S.Z.); cjlsgz@hotmail.com (J.C.); yeyangzhang@126.com (Y.Z.); gzxfl1985@163.com (F.X.); gz8hgpl@126.com (P.G.); tangxp@gzhmu.edu.cn (X.T.); 2 Guangzhou Medical Research Institute of Infectious Diseases, Guangzhou Eighth People’s Hospital, Guangzhou Medical University, Guangzhou 510440, China; 3 Clinical Laboratory, Guangzhou Eighth People’s Hospital, Guangzhou Medical University, Guangzhou 510440, China; ojy907@126.com (J.O.); daiyouchao@gzhmu.edu.cn (Y.D.)

**Keywords:** *Talaromyces marneffei*, aspartic protease, genome-wide identification, expression pattern, stress response

## Abstract

Aspartic proteases (APs), a class of proteolytic enzymes involved in protein maturation, degradation, and signaling, are known to contribute to fungal virulence and pathogenicity. However, the *AP* gene family in *Talaromyces marneffei* (*T. marneffei*), a dimorphic opportunistic pathogenic fungus, has not yet been functionally analyzed. In this study, we identified 27 *AP* genes from the *T. marneffei* genome, and the encoded APs retained conserved domains and exhibited similar motifs and structural properties crucial for catalytic activity. Phylogenetic and collinearity analyses found that *TmAPs* were most recently homologous to *Aspergillus* gene families, with both tandem and segmental duplications contributing to their expansion. Expression patterns, combined with RNA-sequencing data, revealed the specialized roles of *TmAP1* and *TmAP2* in the dimorphic conversion between the yeast and mycelial phases. Protein–protein interaction network analysis uncovered links to cell fusion, mitochondrial function, and programmed cell death. Under abiotic stress conditions, several *TmAP* genes displayed significant transcriptional changes, implying their involvement in short-term adaptation and stress responses. This study provides the first comprehensive and systematic analysis of the *AP* gene family in *T. marneffei*, highlighting their potential biological roles in fungal development, dimorphic conversion, and stress adaptation. Our findings offer valuable insights into further functional characterization of *AP* genes in *T. marneffei* and may facilitate the development of novel therapeutic targets and intervention strategies against *T. marneffei* infection.

## 1. Introduction

The *Talaromyces marneffei* (*T. marneffei*), previously identified as *Penicillium marneffei*, is an important intracellular opportunistic pathogen mainly prevalent in Southeast Asia and southern China [1,2]. It is a typical thermally dimorphic pathogenic fungus, capable of growing as a mycelial mold at 25 °C and transitioning into a yeast form at 37 °C [3]. *T. marneffei* has been increasingly recognized as an emerging human fungal pathogen that causes a fatal disseminated systemic mycosis known as *talaromycosis* in both immunocompetent and immunocompromised individuals, though it is most prevalent in patients with HIV/AIDS or other immune impairments [4,5]. *T. marneffei* infection in patients with HIV has been found to be decreasing with improved access to antiretroviral therapy and antifungal drugs. However, the incidence of *talaromycosis* has recently increased among individuals with secondary immunodeficiency without HIV infection [6]. In high-risk regions of Vietnam, northern Thailand, and southern China, the proportion of HIV hospital admissions with *T. marneffei* infection is up to 9–18%, and the mortality rate among individuals with HIV with *talaromycosis* remains up to 30% despite antifungal therapy [7,8,9]. To date, *talaromycosis* has emerged as the leading cause of HIV-associated death, especially in patients with advanced HIV disease in the above endemic regions. Despite high morbidity and mortality, the pathogenicity and underlying molecular mechanisms of *T. marneffei* infection causing human disease are still poorly understood.

Aspartic proteases (APs), otherwise known as aspartyl peptidases, are crucial proteolytic enzymes ubiquitously distributed across various organisms, including prokaryotes, eukaryotes and retroviruses [10]. These enzymes and their isoenzymes typically form a monomer containing two aspartic acid residues within the conserved Asp-Thr/Ser-Gly motifs, which are essential for their proteolytic activity [11]. APs are involved in numerous important biological processes and play significant roles in morphogenesis, host invasion, nutrition, metabolism, cellular function, and immunity. Recently, APs have been found to act as important virulence agents and key factors implicated in the virulence and pathogenicity of many emerging and established pathogenic microorganisms, especially disease-causing human fungal pathogens [12,13,14]. These hydrolytic enzymes are critical for both fungal physiological processes and host–fungus interactions and may serve essential functions during various stages of fungal infection and pathogenesis, such as adhesion [15,16,17], biofilm formation [18,19], hyphal development [20,21], dimorphic switching [22,23], host invasion, and immune evasion [24,25]. For instance, Kim et al. [26] reported that the secreted aspartyl proteinase SAPA3 emerged as the major AP in *Candida auris* and is involved in biofilm formation. Deletion of sapa3 resulted in diminished virulence, underscoring the role of AP activity as a significant virulence factor contributing to fungal pathogenicity. In *Candida glabrata*, the yapsin family of aspartyl proteases has been found to be critical for the regulation of pH [27], vacuole [28], and glucose homeostasis [29]. A study on *Paracoccidioides brasiliensis* demonstrated that the addition of Pepstatin A, an inhibitor of aspartic proteases, caused a delay in the mycelium-to-yeast dimorphic switching [22]. Overexpression of SAP2 significantly influenced hyphal growth and altered the invasion and adhesion abilities of *Candida albicans* in A431 cells compared to the wild-type strain [30]. Furthermore, it has been proposed that, during host invasion, fungal APs are involved in the degradation of various structural proteins as well as several extracellular matrix proteins. Previous studies have found that certain APs, such as Sap2p [31,32], Sap5p [33], and SAP1 [34], participate in the degradation of host structural proteins to facilitate initial infection and subsequent propagation. In addition, the role of APs in modulating fungal defense strategies against the host immune system has also been extensively documented. In *C. albicans*, the extracellular SAP6 can attenuate the reactive oxygen species (ROS) production through proteolytic damage to NADPH oxidase, thereby impairing neutrophil cellular defense functions [35]. Sequence variation in SAP2 enhances fungal virulence by blocking complement activation and inducing a macrophage M2-like phenotype switch, promoting potent immunosuppression of T cell responses [36]. Moreover, the aspartyl proteases (Yapsins) in *C. glabrata* were found to target Arpc1B, an actin nucleator complex protein, leading to actin disassembly and reduced epithelial cell neutrophil signaling [37]. Overall, the AP proteins of fungi are important molecules that play significant roles in fungi growth, development, and responses to environmental changes.

To date, vaccination and immunotherapy against opportunistic infections still remain a challenge. Recent data demonstrate the prophylactic and therapeutic potential of the antigenic protein AP or AP-specific antibodies against fungal infections. Vernel-Pauillac et al. [13] discovered that mice developing antibodies specifically against Pep1p, an aspartic protease secreted by *Cryptococcus neoformans*, showed significantly improved survival due to enhanced phagocytosis and inhibited fungal multiplication. Major aspartyl peptidase 1 (May1), a secreted protease of *C. neoformans*, has been identified as a potential target for developing active drugs against both fungal and retroviral aspartic proteases [38]. Furthermore, previous studies have evidenced the promising therapeutic potential of the vaccine PEV7 for recurrent vulvovaginal candidiasis (RVVC), which is derived from recombinant *C. albicans* Sap2 [39,40]. Together, these findings suggest that APs can be exploited for vaccination or immunotherapy to improve the host defense and stimulate protective immune responses during fungal infections.

Although aspartic proteases have been reported in numerous fungal species, research on the identification, structural characterization, and comparative evolution of this AP gene family using recently updated genome information is not completely understood in *T. marneffei*. In the current study, an in-depth investigation of the *T. marneffei* AP gene family was conducted through a combination of in silico analysis and experimental approaches. Here, we performed a genome-wide identification and characterization of the AP gene family in *T. marneffei* and systematically analyzed the physicochemical traits, subcellular localization, chromosomal distribution, gene structure, conserved motifs, evolutionary relationships, and synteny relationships. By employing an integrated approach combining RNA sequencing (RNA-seq) and quantitative reverse transcription polymerase chain reaction (RT-qPCR), we subsequently elucidated the expression patterns of these AP homologs under different culture conditions and abiotic stresses. Collectively, these findings expand our understanding of the evolutionary relationships and functional characteristics of the AP family in *T. marneffei*. The present study also provides new insights for further research on the pathogenicity and molecular mechanisms of *T. marneffei* infection, as well as vaccine development.

## 2. Materials and Methods

### 2.1. Fungal Strains and Cell Culture

A total of 18 *T. marneffei* isolates with culture-documented infection were included in the present study (Table S1). All isolates were identified by morphological characteristics, nuclear ribosomal DNA internal transcribed spacer region (ITS) sequence analysis, or matrix-assisted laser desorption ionization time-of-flight mass spectrometry (MALDI-TOF MS, Bruker, karlsruhe, Germany) before being preserved in the Infectious Disease Center, Guangzhou Eighth People’s Hospital. For strain revival, porcelain bead stocks were inoculated into fresh Sabouraud’s Dextrose Broth (SDB, Vazyme, Nanjing, China) medium and cultured in incubator shakers at 37 °C for 3–5 days. *T. marneffei* conidia were activated at 28 °C on Sabouraud Dextrose Agar (SDA, Vazyme, Nanjing, China) for 5–7 days and subsequently grown on Potato Dextrose Agar (PDA, Vazyme, Nanjing, China) medium for 7 days. Conidia were harvested by flooding the surface of the culture medium with phosphate-buffered saline (PBS, Vazyme, Nanjing, China), suspended in PBS, and counted using a hemocytometer. The strains were then cultured on PDA at 28 °C for spore germination and hyphal growth, or at 37 °C for morphological transformation.

For stress treatments, the SDA plates were supplemented with either 500 mM NaCl (Sangon Biotech, Shanghai, China) (to simulate salt stress) or 500 mM sorbitol (Sangon Biotech, Shanghai, China) (to simulate osmotic stress). Oxidative stress was induced by adding 5 mM H_2_O_2_ (Sangon Biotech, Shanghai, China), and antifungal stress was simulated by adding 10 μg/mL amphotericin B (Sangon Biotech, Shanghai, China). All samples were cultured at 37 °C and collected 24 h after treatment initiation. The normal growth group (CK) was maintained under identical conditions without any supplementation.

RAW264.7 murine macrophage cells (Fuheng Biotech, Shanghai, China) were cultured in Dulbecco’s Modified Eagle Medium (DMEM, Sangon Biotech, Shanghai, China) supplemented with 10% fetal bovine serum (FBS, Vazyme, Nanjing, China) and 1% penicillin–streptomycin (Solarbio, Beijing, China). The cells were maintained at 37 °C in a humidified incubator containing 5% CO_2_ and replenished with fresh medium every 3 days. M1 polarization of macrophages was induced by treatment with 500 ng/mL lipopolysaccharide (LPS, Solarbio, Beijing, China) for 24 h. Macrophages were infected with *T. marneffei* conidia at a multiplicity of infection (MOI) of 5 at 37 °C for 24 h, and the fungi were collected for subsequent experiments.

### 2.2. Identification and Characterization of AP Family Genes in T. marneffei

To identify AP genes in *T. marneffei*, we employed a comprehensive approach using both HMMER search and BLASTP (https://blast.ncbi.nlm.nih.gov/Blast.cgi?PAGE=Proteins; accessed on 5 December 2025). In brief, the hidden Markov model (HMM) profile for the Asp domain (PF00026) from the Pfam database (http://pfam.xfam.org/; accessed on 5 December 2025) was used as a query to search the *T. marneffei* genome (obtained from the NCBI GenBank assembly version: ASM4612747v1, accession number: GCA_046127475.1) by HMMER v3.4. A SEED file was generated following the ‘hmmbuild’ command, and ‘hmmsearch’ was used to screen for candidate AP gene family members in the whole-genome protein sequence file. Next, we conducted a local BLASTP (score value ≥ 100, E-value ≤ 1 × 10^−10^) search to compare the SEED file with the protein sequence database of *T. marneffei*. For validation of candidate sequences, the structural domains of hypothetical proteins were verified using two databases: the NCBI Conserved Domain Database (CDD) (https://www.ncbi.nlm.nih.gov/cdd; accessed on 12 December 2025) and SMART (https://smart.embl.de/; accessed on 12 December 2025). After eliminating redundant and structurally incomplete domain sequences, candidate genes corresponding to protein sequences containing the AP structural domain were considered members of the AP gene family. Characterization of the physicochemical properties of TmAP proteins, including protein length, molecular weight (MW), theoretical isoelectric point (pI), instability index (II), aliphatic index (AI), grand average hydropathicity (GRAVY) and others, were calculated using the ExPASy ProtParam online resources (https://web.expasy.org/protparam/; accessed on 16 December 2025). DeepTMHMM v1.0 was utilized to predict transmembrane helices in proteins (https://services.healthtech.dtu.dk/services/DeepTMHMM-1.0/; accessed on 18 December 2025).

### 2.3. Sequence Alignment and Phylogenetic Tree Construction

The multiple sequence alignments, including TmAPs and their homologs from other species, were performed using the Clustal Omega online program with default parameters (https://www.ebi.ac.uk/jdispatcher/msa/clustalo; accessed on 10 January 2026), followed by manual modification and visualization with the GeneDoc v2.7 software package.

To elucidate the evolutionary relationships between different species, the AP genes’ amino sequences of *Aspergillus fumigates*, *Aspergillus oryzae*, *Aspergillus niger*, *Candida albicans*, *Saccharomyces cerevisiae*, and *Cryptococcus neoformans* were obtained from the NCBI genebank database. All protein sequences were subjected to MEGA7.0 software for alignment by using ClustalW with default parameters. A root-free evolutionary tree was then constructed through the neighbor-joining (NJ) method with the best-fit substitution Poisson model. The bootstrap value was set to 1000 replicates. The tree was finally modified and visualized using the iTOL online tool (https://itol.embl.de/; accessed on 12 January 2026).

### 2.4. Chromosomal Distribution, Genomic Duplications, and Synteny Analysis

Using the NCBI database, the genome annotation files of *T. marneffei* were obtained, and the genomic positions of *TmAP* genes on chromosomes and their corresponding physical information were identified using TBtools-II v2.423 software based on gene annotations [41]. The chromosomal localization of *TmAP* genes was visually represented using the Gene Loaction Visualize (advanced) program. Inter- and intra-species collinearity of *TmAP* genes between *T. marneffei* and four other species was defined using the One Step MCScanX method within TBtools software. To investigate the evolutionary constraints acting on each pair of *TmAP* genes, the synonymous substitution rate (*K*s) and non-synonymous substitution rate (*K*a) were calculated using DnaSP v6.12.3 software. Selective pressure on *TmAP* genes during evolution was assessed by analyzing the *K*a/*K*s ratios of collinear gene pairs within the *TmAP* gene family. A Ka/Ks ratio significantly greater than 1, less than 1, or equal to 1 indicates positive, negative, or neutral selection, respectively. Potential tandem and segmental duplication events involving *TmAP* genes were analyzed using TBtools with default parameters.

### 2.5. Gene Structure and Conserved Motif Analyses of TmAP Family Members

The exon–intron distribution of *TmAP* genes was analyzed using the TBtools software based on the GFF annotation files of *T. marneffei* obtained from NCBI genome database. The structural domains of TmAP proteins were predicted using the NCBI Conserved Domain Database (https://www.ncbi.nlm.nih.gov/cdd/; accessed on 20 January 2026). Conserved motifs in the TmAP proteins were analyzed using the Multiple Em for Motif Elicitation (MEME) v5.5.9 (https://meme-suite.org/meme/tools/meme; accessed on 22 January 2026), with a maximum number of motifs of 10 with default parameters. All results were visualized by using TBtools.

### 2.6. Prediction of 3D Structure and Subcellular Localization Analysis

Phyre2.0 server (https://www.sbg.bio.ic.ac.uk/phyre2/html/page.cgi?id=index; accessed on 5 February 2026) was used to estimate the three-dimensional (3D) protein structure, and the resulting pdb files were visualized with PyMOL v3.0.3 software. Protein secondary structure prediction was conducted using the SPOMA software, available at (https://npsa.lyon.inserm.fr/cgi-bin/npsa_automat.pl?page=/NPSA/npsa_sopma.html; accessed on 11 February 2026). The subcellular localization of the *TmAP* family genes was analyzed using three online prediction tools: ProtParam (https://web.expasy.org/protparam/; accessed on 15 February 2026), WoLF-PSORT (https://www.genscript.com/wolf-psort.html?src=leftbar; accessed on 15 February 2026), and Cell-PLoc (http://www.csbio.sjtu.edu.cn/bioinf/Cell-PLoc/; accessed on 16 February 2026).

### 2.7. Total RNA Extraction, Library Construction and Illumina Sequencing

Total RNA was extracted using Trizol reagent kit (Invitrogen, Carlsbad, CA, USA) according to the manufacturer’s protocol. RNA quality was assessed using an Agilent 2100 Bioanalyzer (Agilent Technologies, Palo Alto, CA, USA) and verified by RNase-free agarose gel electrophoresis. mRNA was isolated using oligo (dT) beads (Epicentre, Madison, WI, USA), and then the enriched mRNA was fragmented into short fragments using RNA fragmentation buffer. The fragmented RNA was then reverse transcribed into cDNA using the NEBNext^®^ Ultra^™^ II RNA Library Prep Kit for Illumina^®^ (NEB#7530, New England Biolabs, Ipswich, MA, USA). The purified double-stranded cDNA fragments were subjected to end repair, ‘A’ base adding, and ligation to Illumina sequencing adapters. The ligation reaction was purified with AMPure XP Beads (1.0×) (Beckman Coulter, Brea, CA, USA), and the obtained fragments were size-selected by agarose gel electrophoresis followed by polymerase chain reaction (PCR) amplification. Library quality was assessed using the Agilent 2100 Bioanalyzer system, and the concentration was measured with a Qubit^®^ 2.0 Fluorometer (Invitrogen, Carlsbad, CA, USA). The resulting cDNA libraries were deep-sequenced on an Illumina NovaSeq 6000 platform (Illumina, San Diego, CA, USA) by Gene Denovo Biotechnology Co. (Guangzhou, China).

### 2.8. Quantitative Real-Time PCR (RT-qPCR) Analysis

Total RNA was extracted using the E.Z.N.A.^™^ Fungal RNA Kit (Omega, Norcross, GA, USA, Cat. No:R6834) according to the manufacturer’s instructions. The A_260/280_ and A_260/230_ ratios and RNA concentration were measured using a NanoDrop one Spectrophotometer (Thermo Fisher, Waltham, MA, USA). For first-strand cDNA synthesis, approximately 1 µg of total RNA from each sample was reverse transcribed in a 20 µL reaction system using the PrimerScript^TM^ RT reagnet Kit with gDNA Eraser (Takara, Dalian, China). All cDNA samples were diluted to the same concentration to serve as a template for subsequent RT-qPCR analysis.

Transcript levels were quantified using TB Green^®^ Premix Ex Taq™ II (Tli RNaseH Plus) (Takara, Dalian, China) on a CFX96 Real-Time PCR Detection System (Bio-Rad, Hercules, CA, USA). Each reaction mixture consisted of 5 µL of Premix Ex TaqII (2×), 0.5 µL of upstream primer (10 µM), 0.5 µL of downstream primer (10 µM), 1 µL of cDNA template, and 3 µL of ddH_2_O in a final reaction volume of 10 μL. The standard thermal cycle procedure was as follows: 2 min at 95 °C; 40 cycles of 15 s at 95 °C; and 20 s at 60 °C, with melting curve detection from 65 °C to 95 °C. Relative expression levels were calculated using the 2^−∆∆Ct^ method [42], with constitutively expressed *β-Actin* as the reference gene. Three biological replicates and three technical replicates were included for each sample. Results were presented as means ± standard error (SE). All primers were designed using the Primer-BLAST program and are listed in Supplementary Table S2.

### 2.9. RNA-Seq Analysis, Expression Profile, Gene Ontology and KEGG Annotation Analysis of TmAPs

Raw RNA-seq reads were processed using FastQC (v0.11.9) for initial quality assessment. Adapter trimming and quality filtering were performed using fastp (v0.18.0) with the following parameters: (1) removing reads containing adapters; (2) removing reads containing more than 10% of unknown nucleotides (N); and (3) removing low-quality reads containing more than 50% of low-quality (Q-value ≤ 20) bases. Clean reads were aligned to the *T. marneffei* reference genome (NCBI RefSeq assembly ASM4612747v1, accession number GCA_046127475.1, strain ATCC18224) using HISAT2 (v2.2.4) with default parameters for “-rna-strandness RF”. Transcript quantification was performed using StringTie (v1.3.1) in a reference-based approach. FPKM (Fragments Per Kilobase of transcript per Million mapped fragments) values were calculated using RSEM (v1.3.1) software, based on the formula: FPKM = (read counts × 10^9^)/(total mapped fragments × transcript length in bp). Differential expression analysis was performed using DESeq2 (v1.30.1). Genes with the parameter of false discovery rate (FDR) below 0.05 and absolute fold change ≥ 1 were considered differentially expressed. Expression of each TmAP gene was quantified in FPKM. The expression values were log-transformed using Log2 (FPKM + 1) and subsequently normalized via Z-score normalization. An expression profile heat map was visualized with the HeatMap illustrator tool using TBtools-II Software. The genome-wide functional annotation of *T. marneffei* was performed using the eggNOG-mapper online tool (https://hpc.nih.gov/apps/eggNOGmapper.html; accessed on 17 February 2026) with default parameters, and the resulting annotation file was subsequently analyzed with TBtools for GO (Gene Ontology) and KEGG (Kyoto Encyclopedia of Genes and Genomes) enrichment analysis.

### 2.10. Protein–Protein Interaction Network Analysis of TmAPs

To better understand the possible interactions of TmAPs with other proteins, network analysis of protein–protein interactions (PPIs) was constructed using the STRING v12.0 program (https://cn.string-db.org/; accessed on 25 February 2026), with a high confidence parameter of ≥0.700 and a false discovery rate (FDR) stringency of 5%. The interaction network was searched using the “Proteins by sequences” module of STRING and subsequently visualized by Cytoscape v3.10.2.0 software.

### 2.11. Statistical Analysis

All experiment data were presented as the mean ± standard error (SE). Normal distribution of the data and variance homogeneity were assessed using Shapiro–Wilk and Levene’s tests, respectively. The two-tailed unpaired Student’s *t*-test (between two groups) and one-way ANOVA with Tukey’s multiple-comparison test (more than two groups) were performed for statistical analysis. All graphs were generated using GraphPad Prism v8.0.1. *p* values of less than 0.05 were considered statistically significant. * *p* < 0.05, ** *p* < 0.01, and *** *p* < 0.001.

## 3. Results

### 3.1. Identification and Physicochemical Properties of the AP Genes in T. marneffei

A total of 27 candidate *AP* genes were ultimately identified throughout the whole genome of *T. marneffei* (Table S3). The physicochemical parameters of these identified proteins were subsequently analyzed in detail (Table S4). According to the analysis results, the molecular weight (MW) of the TmAPs was between 41.3 and 45.84 kDa, and the theoretical isoelectric point (pI) was between 4.44 and 9.22. The instability index varied from 10.77 to 40.59, with 25 proteins being classified as stable (instability index < 40). The grand average hydropathy index (GRAVY) value was between −0.406 and 0.167, and most GRAVY values of TmAPs were less than 0, indicating their hydrophilic nature. Subcellular localization predictions using WoLF PSORT confirmed that 26 out of 27 TmAP proteins were likely located primarily in the extracellular, and one out of 27 was located in the nucleus, which was consistent with the predictive subcellular localization results obtained from Cell PLoc.

### 3.2. Multiple Sequence Alignment and Protein Structure Prediction of TmAPs

**Figure 1:**
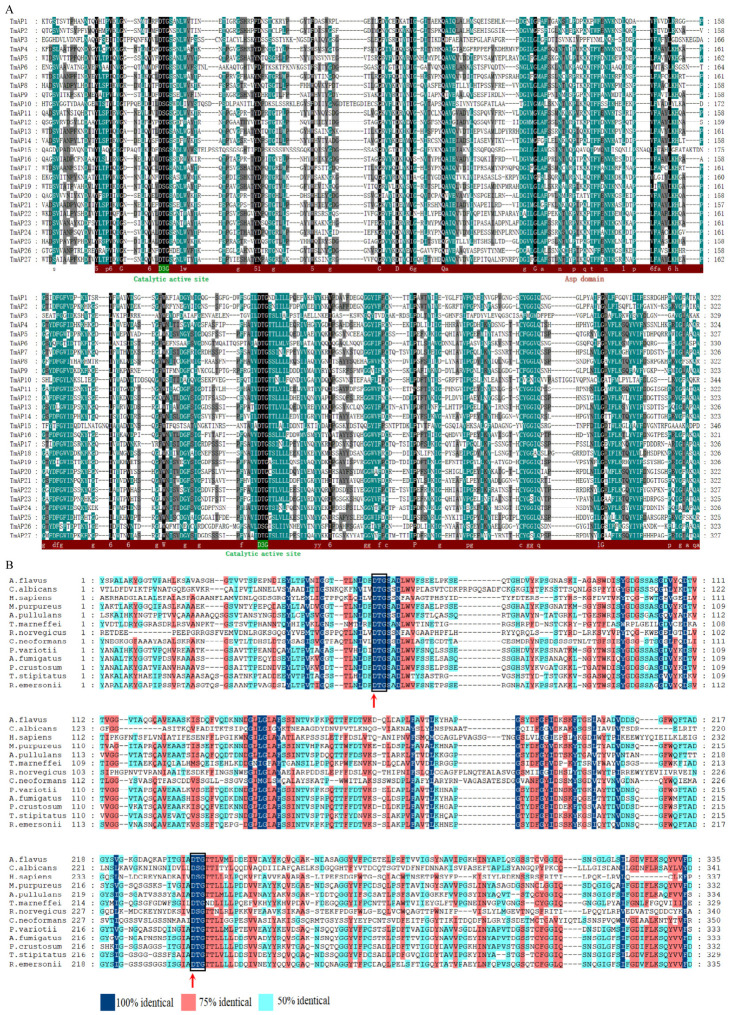
Multiple sequence alignment of the TmAP proteins. (**A**) The amino acid sequences underwent multiple sequence alignment derived from each *TmAP* gene. Sequence identity and similarity were represented by black, green, and gray letters, respectively. The background of amino acid residues is based on the conserved percent (black indicates 100%, green indicates 75%, and gray indicates 50%). The red shadow indicates the Asp conserved domain, and the green shadows represent catalytic active sites. (**B**) Multiple sequence alignment of the AP proteins in *Talaromyces marneffei*, *Aspergillus flavus*, *Aspergillus fumigates*, *Aureobasidium pullulans*, *Candida albicans*, *Cryptococcus neoformans*, *Homo sapiens*, *Monascus purpureus*, *Paecilomyces variotii*, *Penicillium crustosum*, *Rasamsonia emersonii*, *Rattus norvegicus*, *Talaromyces stipitatus*. The background of amino acid residues is based on the degree of conservation (dark blue indicates 100%, red indicates 75%, and light blue indicates 50%). The DT/SG domains are displayed as black boxes. The red arrows represent aspartate residues.

Comparing all TmAPs, the BLAST algorithm against NCBI confirmed sequence identity and similarity ranging from 50% to 75%. Using the Clustalw program, the amino acid sequences of the identified AP homologs were subjected to multiple sequence alignment. As shown in Figure 1A, the comparison result revealed that all TmAP proteins were found to have a complete aspartyl protease (Asp) domain (PF00026), which contained two invariant aspartic acid catalytic residues positioned within the hallmark motifs Asp-Thr/Ser-Gly (DT/SG). In addition, to further understand the evolutionary characteristics, the homologous sequences of AP proteins utilizing corresponding data information derived from other species were aligned. The multiple sequence alignment suggested that the AP protein sequence in *T. marneffei* is analogous to those seen in other species (Figure 1B), and the catalytic active sites within their functional domains are conserved in these genes. These findings exhibited high sequence identity and conserved domains among different species, implying the same biological function during evolution.

The secondary structure of TmAPs was predicted using the SPOMA server with default parameters, as depicted in Supplementary Table S5. The secondary structures of TmAPs were primarily composed of random coil, followed by extended strand and alpha helix, with the proportions of these three structural components being roughly identical across all TmAPs. Tertiary structure prediction of AP homologous proteins from *T. marneffei* was conducted using the Phyre v2.2 workspace and showed a high degree of similarity (Table S5). Notably, the typical conservative feature seen in all examined proteins is an alpha/beta monomer composed of two asymmetric lobes, each contributing an active site, with the catalytic aspartic acid residue located in the groove formed by the two lobes. Furthermore, the sequence identity searched by using the SWISS-MODEL server observed in this study varied ranging from 75.76% to 100%, with the maximum sequence identity found in TmAP6, TmAP7, TmAP10, TmAP20, TmAP21, and TmAP24 and the minimum found in TmAP15. The GMQE values of all TmAPs were higher than 0.75, with a range from 0.80 to 0.89, indicating that the homologous modeling for the TmAP proteins was highly accurate.

### 3.3. Chromosomal Location of AP Genes in T. marneffei

**Figure 2:**
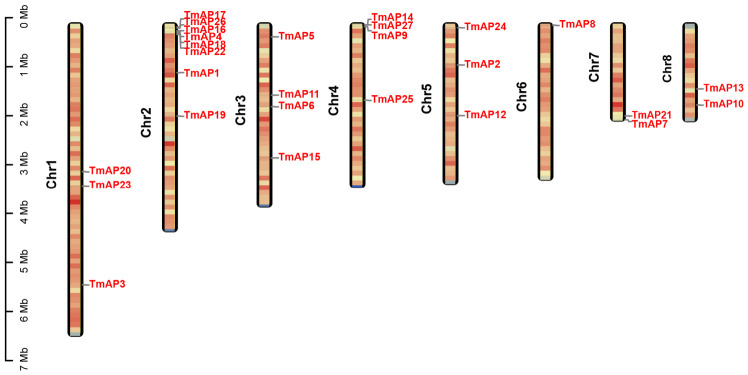
Schematic diagram showing the chromosomal location and distribution of *AP* genes in *T. marneffei*. The colored rectangular bars represent the chromosomes of *T. marneffei*. A total of 27 identified AP homolog genes are mapped to the eight chromosomes. The chromosome number is displayed at the left of each bar. The gene names are indicated in red on the right side. The scale of the chromosome located on the left panel is in millions of bases (Mb).

Based on the annotation information of the *T. marneffei* genome, the genomic chromosomal distribution of the identified *TmAP* genes was mapped to their respective chromosomal positions using the TBtools software with the Gene Location Visualize (Advanced) option. As shown in Figure 2, the results of visual analysis revealed that the 27 *TmAP* genes were unevenly spread across the eight chromosomes, and the positioning of *AP* genes on the chromosomes of *T. marneffei* was predominantly distributed in the top and middle sections. Among them, Chromosome 2 (Chr2) contained the largest *AP* genes at eight (29.63%), while the lowest number, one *AP* gene (3.70%), was present on Chr6. Chr3 and 4 each contained four genes (14.81%), Chr1 and 5 each contained three genes (11.11%), and Chr7 and 8 each contained only two genes (7.41%).

### 3.4. Gene Structure, Conserved Domain and Motif Analysis of TmAP Gene Family

**Figure 3:**
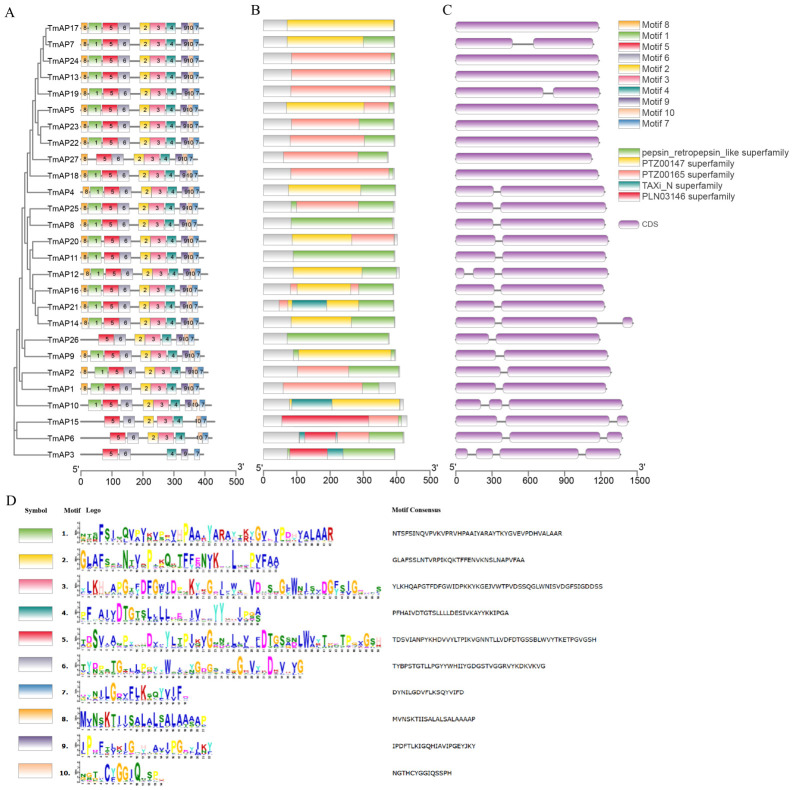
Gene structure, motif, and conserved domain analysis of the TmAP gene family. (**A**) Distribution of motifs within each TmAP protein. (**B**) Conserved domains of TmAPs predicted by batch CD-search in NCBI. (**C**) Gene structure of *TmAPs*; lines indicate introns and boxes represent exons. (**D**) The motif symbols along with motif consensus represent conserved motifs: 1–10.

To gain further insights into the motif composition of the AP family in *T. marneffei*, the conserved motifs of 27 TmAP proteins were analyzed by performing a MEME search, and a total of 10 separate motifs (named from motif 1 to motif 10) were identified (Figure 3A,D). The number of motifs recognized in all TmAPs exhibited a range of five to ten, and these motifs varied in size from 14 to 50 AA. Among them, motifs 2, 3, and 10 were detected in 26 proteins except TmAP3, motif 9 was identified in 25 proteins except TmAP6 and TmAP15, while motifs 1 and 8 were identified in 22 proteins. Significantly, we also found that motifs 4–7 were observed across all 27 TmAP members, and the ordering of the four motifs was consistently 5, 6, 4, and 7. Motifs 5 and 4 were highly conserved and shown to contain the consensus motif DTGs, which encodes an aspartic acid residue crucial for catalytic activity. According to the results of conserved domain analysis obtained via SMART, all TmAP proteins contained a conserved Pfam Asp domain (PF00026). In addition, by further searching against the NCBI Conserved Domain Database (CDD), all selected protein sequences were classified as belonging to the pepsin/retropepsin-like superfamily (Figure 3B), confirming the reliability of the domain architecture analysis. All TmAP proteins contain an N-terminal signal peptide but do not have transmembrane regions, except for TmAP12, TmAP15 and TmAP26, implying that most of them are defined as secretory proteins.

Next, we investigated the gene structure of *TmAPs* to gain a deeper comprehension of the correlation between the structural similarity and evolutionary function of the *TmAP* gene family. Comparative analysis of exon–intron patterns, as illustrated in Figure 3C, revealed that TmAP genes varied in both the number and positional arrangement of their exons (ranging from 1 to 4) and introns (ranging from 0 to 3). For instance, TmAP1 and TmAP2, which share introns at identical positions, are more likely to be evolutionarily closer to each other than to TmAP7 and TmAP19, which harbor introns at different positions. Similarly, TmAP6 and TmAP15, also possessing introns at the same positions, appear more closely related to each other than to TmAP10 and TmAP12, which harbor introns at different positions. When intron positioning was used as a phylogenetic marker, the resulting patterns corroborated previously established relationships. Genes with similar intron numbers and distributions tend to cluster within the same major lineages, which underscores their evolutionary conservation.

### 3.5. Evolutionary Analysis of the TmAP Gene Family

**Figure 4:**
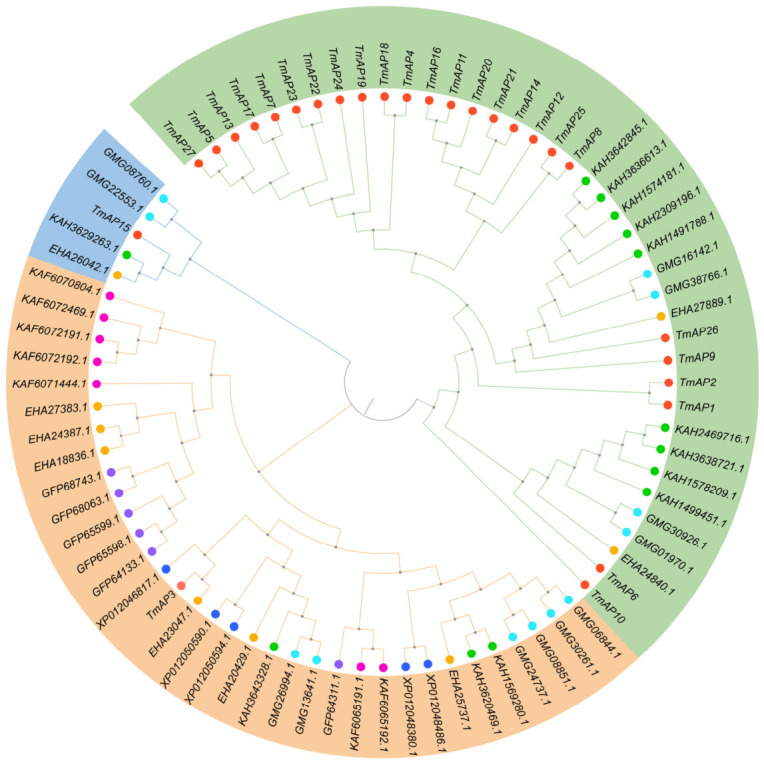
Phylogenetic analysis of AP homolog proteins derived from *T. marneffei* and other fungal species. An unrooted neighbor-joining tree was constructed using 79 aligned proteins from 27 *Talaromyces marneffei* (TmAP1-TmAP27), 13 *Aspergillus fumigates* (KAH1491788.1-KAH3643328.1), 12 *Aspergillus oryzae* (GMG01970.1-GMG38766.1), nine *Aspergillus niger* (EHA18836.1-EHA27889.1), seven *Candida albicans* (KAF6065191.1-KAF6072469.1), six *Saccharomyces cerevisiae* (GFP64133.1-GFP68743.1), and five *Cryptococcus neoformans* (XP012046817.1-XP012050594.1) in MEGAv7.0, with bootstrap values calculated from 1000 replicates. The AP proteins were clustered into three groups, each represented by a distinct color.

Phylogenetic analysis plays a crucial role in elucidating the evolutionary relationships of genes among species. In order to further explore the evolutionary lineage and functional characteristics of the *TmAP* gene family, an unrooted phylogenetic tree of AP genes in seven species including *T. marneffei* was constructed using the neighbor-joining method (Figure 4). A total of 79 AP proteins from different species were analyzed, including 27, 13, 12, nine, seven, six, and five from *T. marneffei* (Tm), *Aspergillus fumigates* (Af), *Aspergillus oryzae* (Ao), *Aspergillus niger* (An), *Candida albicans* (Ca), *Saccharomyces cerevisiae* (Sc), and *Cryptococcus neoformans* (Cn), respectively. Phylogenetic analysis suggests that the AP proteins cluster into three groups. Among them, the AP proteins in *T. marneffei* are more strongly homologous to *Aspergillus fumigatesand*, *Aspergillus niger*, and *Aspergillus oryzae* than to *Cryptococcus neoformans*, *Candida albicans*, and *Saccharomyces cerevisiae*. The outcome of phylogenetic relationship evaluation confirmed that the *AP* genes of *T. marneffei* and *Aspergillus* spp. shared a high degree of homology, indicating that the protein sequences of these genes may perform similar evolutionary processes and biological functions.

### 3.6. Synteny and Duplication Analysis of the TmAP Family

**Figure 5:**
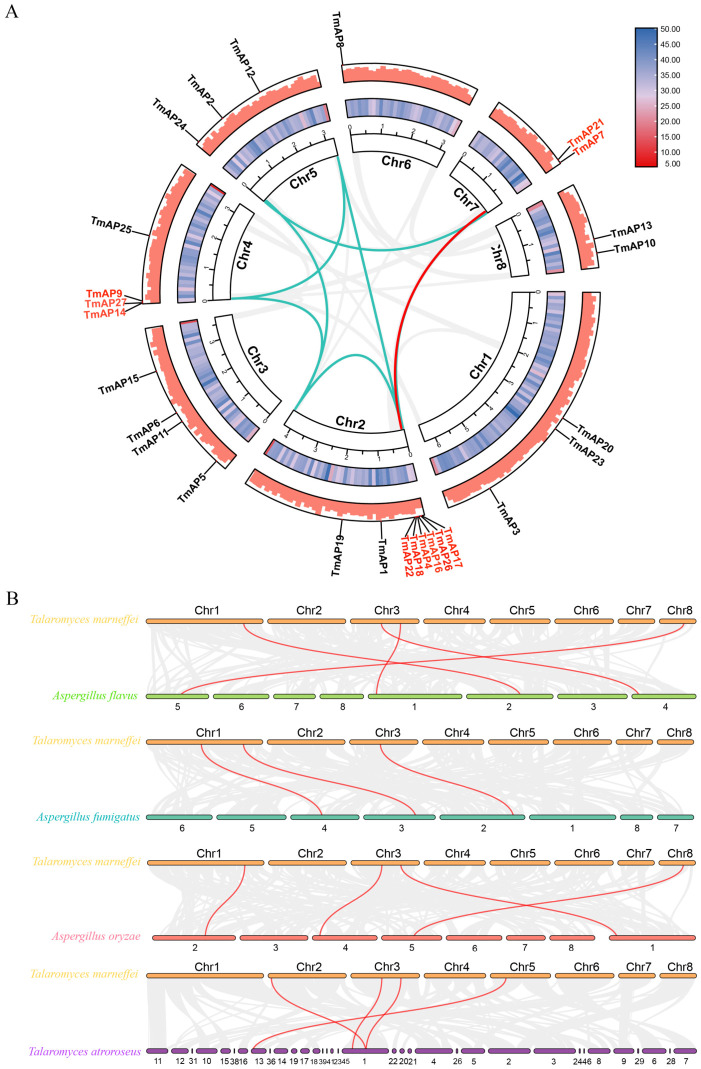
The collinear relationships of *TmAP* genes. (**A**) Collinearity analysis of *AP* genes in *T. marneffei.* The chromosomal distribution of *TmAPs* and the interactions between chromosomes are shown as circles. Chromosomes are represented by inner ring boxes, with gene names surrounding the boxes and the chromosome number shown beside each chromosome. Gene density in each chromosome is represented by a heat map and bar plot. The syntenic blocks in the *T. marneffei* genome are shown by the gray lines in the background. The red lines represent syntenic *TmAP* gene pairs, whereas the green lines indicate homologous gene pairs consisting of one *TmAP* and another gene (not a *TmAP*). Genes in red font represent tandem repeat genes. (**B**) Inter-species collinearity analysis of *AP* genes among *Talaromyces marneffei*, *Aspergillus flavus*, *Aspergillus oryzae*, *Aspergillus fumigatus*, and *Talaromyces atroroseus*. Red lines indicate the orthologous gene pairs of *T. marneffei* and other species.

As a potential source of genetic variation, gene duplication can lead to the expansion of gene family members and facilitate the emergence of new gene families during evolution. Two or more adjacent homologous genes arranged in a head-to-tail orientation within a 200 kb chromosomal region, with no more than one intervening gene, are considered the result of tandem duplication events [43,44]. To gain a deeper comprehension of the duplication activities and evolutionary relationships, an investigation into the collinearity was conducted on both the segmental and tandem duplications within the *TmAP* gene family. The outcomes of intra-species synteny analysis showed that nine gene pairs were identified with segmental duplication events (Figure 5A). Among them, one *TmAP* gene pair was collinear, while the remaining eight gene pairs were collinear with other genes. Notably, several TmAP genes were found to form physical clusters on specific chromosomes, and further analysis confirmed that these clusters correspond to genuine tandem duplication events. In total, we observed eight such events encompassing 11 TmAP genes distributed across chromosomes 2, 4, and 7 (Figure 5A, Table S6). Further examination was conducted to estimate the values of Ka (non-synonymous substitution rate) and Ks (synonymous substitution rate) and the Ka/Ks ratio of the selected homologous gene pairs, aimed to enhance our understanding of the evolutionary constraints on the *TmAP* gene family. The duplicated gene pairs displayed Ka/Ks values ranging from 0.52 to 0.81 (Table S7), implying that the AP gene family in *T. marneffei* has primarily undergone purifying selection under selective pressure during its evolutionary history.

Furthermore, an intergenomic collinearity analysis of AP genes was performed between *T. marneffei* and the other four species, including *Aspergillus fumigatus, Aspergillus flavus*, *Aspergillus oryzae*, and *Talaromyces atroroseus* (Figure 5B). The results of synteny analysis showed that collinear genes were unevenly distributed on different chromosomes in each species, and a total of 15 orthologous gene pairs were found between *T. marneffei* and the other four species. The *TmAP* genes were homologous to genes in other fungi, and syntenic conservation was observed among *A. flavus* (four orthologous gene pairs distributed on Chr5, Chr1, Chr2, and Chr4)*, A. fumigates* (three orthologous gene pairs distributed on Chr4, Chr3, and Chr2), *A. oryzae* (four orthologous gene pairs distributed on Chr2, Chr4, Chr5 and Chr1), and *T. atroroseus* (four orthologous gene pairs distributed on Chr13 and Chr1). Both segmental duplication events and tandem duplication events related to APs can be found in the *T. marneffei* genome, indicating that both duplication processes contributed to the expansion and evolution of the AP family.

### 3.7. Expression Patterns of TmAP Genes and RT-qPCR-Based Validation

**Figure 6:**
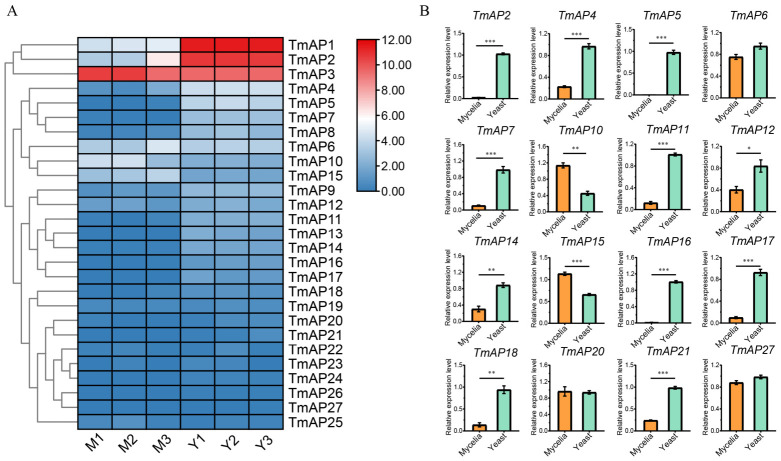
Expression characteristics of *TmAPs.* (**A**) Hierarchical clustering of expression patterns of *TmAPs* in different phases. The expression heat map was constructed using FPKM values from RNA-seq data. The original FPKM values of the TmAP genes were subjected to Log_2_(FPKM + 1) logarithmic transformation. The color scale represents log2 expression values, ranging from blue for low expression levels to red for high expression levels. Y1–Y3 and M1–M3 represent three independent biological replicates of yeast-phase and mycelia-phase cells, respectively. (**B**) Validation of selected *TmAP* genes via RT-qPCR. The * indicates significant differences by *t*-test (*, *p* < 0.05, **, *p* < 0.01, ***, *p* < 0.001).

To investigate the expression profiles of AP genes in the mycelial and yeast phases of *T. marneffei*, the relative expression levels of *TmAP* genes were analyzed based on transcriptome data to generate an expression heat map (Figure 6A). The hierarchical clustering analysis demonstrated that the expression of AP family genes varied between the two phases. Of the 27 identified *TmAP* genes, 22 were found to be highly expressed in the yeast phase at 37 °C compared to the mycelial phase at 28 °C, with *TmAP1* and *TmAP2* showing particularly notable up-regulation. The above results suggest that these *TmAP* genes with increased expression levels are closely involved in the regulation of biphasic transition and/or pathogenic processes during *T. marneffei* infection.

Additionally, in the present study, we conducted validation by subjecting 16 *TmAPs* to RT-qPCR experiments with specific primers to confirm the accuracy of the transcriptome data (Figure 6B). Comparative analysis indicated that the relative transcript levels of the candidate genes were generally consistent with the expression trends obtained from our RNA-seq data. For instance, 11 *TmAPs*, i.e., *TmAP2*, *TmAP4*, *TmAP5*, *TmAP7*, *TmAP11*, *TmAP12*, *TmAP14*, *TmAP16*, *TmAP17*, *TmAP18* and *TmAP21*, were up-regulated in both RNA-seq and RT-qPCR, whereas the other two *TmAPs*, i.e., *TmAP10* and *TmAP15*, were all down-regulated in both RNA-seq- and RT-qPCR-based gene expression analyses. A significant correlation was observed between the gene expression results obtained from RNA-seq data and RT-qPCR analysis, confirming the accuracy and reliability of our findings.

### 3.8. Expression Analysis of TmAP1 and TmAP2 in Various T. marneffei Strains

**Figure 7:**
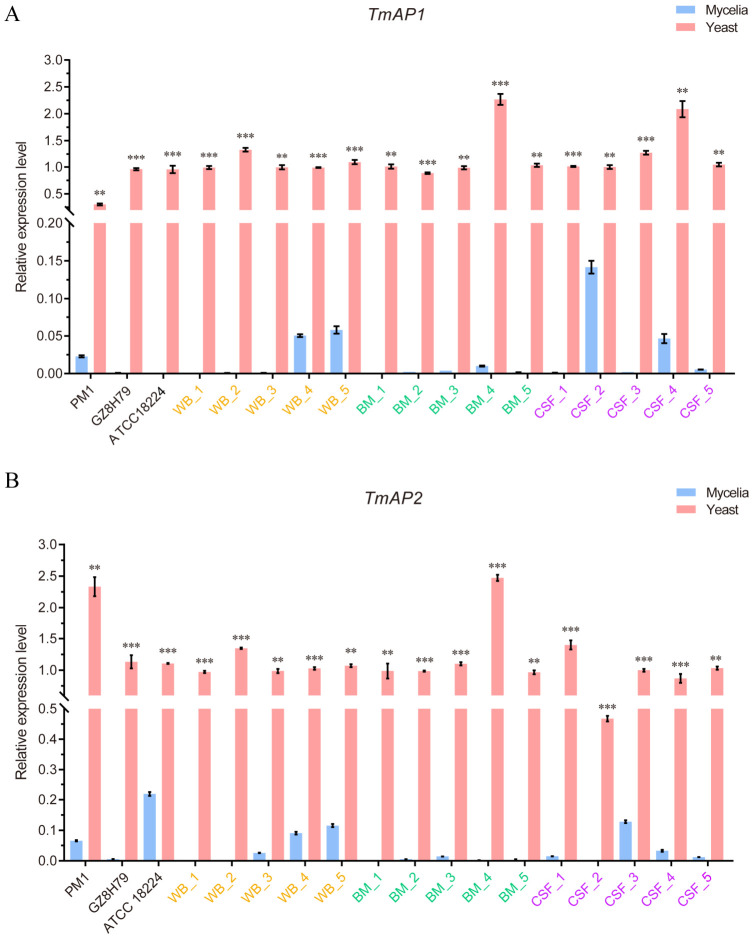
RT-qPCR analysis of *TmAP1* (**A**) and *TmAP2* (**B**) expression in *T. marneffei* strains from various tissues samples. Reference strains: PM1, ATCC18224; clinical isolates were derived from the following sources: whole blood (WB), bone marrow (BM), and cerebrospinal fluid (CSF). ** and *** represent significant differences at the 0.01 and 0.001 levels by Student’s *t*-test, respectively.

When comparing the transcriptome results of *T. marneffei* strain GZ8H79 used in this experiment, it was observed that two *TmAP* genes (*TmAP1* and *TmAP2*) exhibited extraordinarily high expressions in the yeast phase compared to the hypha-inducing condition (*TmAP1*_log_2_(fc) = 6.81 and *TmAP2*_log_2_(fc) = 5.72, respectively). In order to verify the specificity of the expression of these two genes, we collected two commercialized standard strains as well as several sets of clinical isolates separated from the patient’s tissues samples, including whole blood, bone marrow, and cerebrospinal fluid. RNA were extracted from all specimens, reverse transcribed into cDNA, and finally processed by RT-qPCR to calculate the relative expression levels, as evident in Figure 7. Notably, in the present investigation, the expression levels of *TmAP1* (Figure 7A) and *TmAP2* (Figure 7B) were shown to be significantly up-regulated across all treatments in the pathogenic yeast form compared to the mold form. This observation can be attributed to the increasing *TmAPs* transcript levels when the fungus was temperature-induced. Our findings suggest that *TmAP1* and *TmAP2* are closely involved with the thermal-mediated dimorphic conversion process, and that overexpression, knockdown, and knockout of these two genes may contribute to functional exploration of the development, virulence, and pathogenicity of *T. marneffei*.

### 3.9. Gene Ontology (GO) Annotation and KEGG Enrichment Analysis of TmAPs

**Figure 8:**
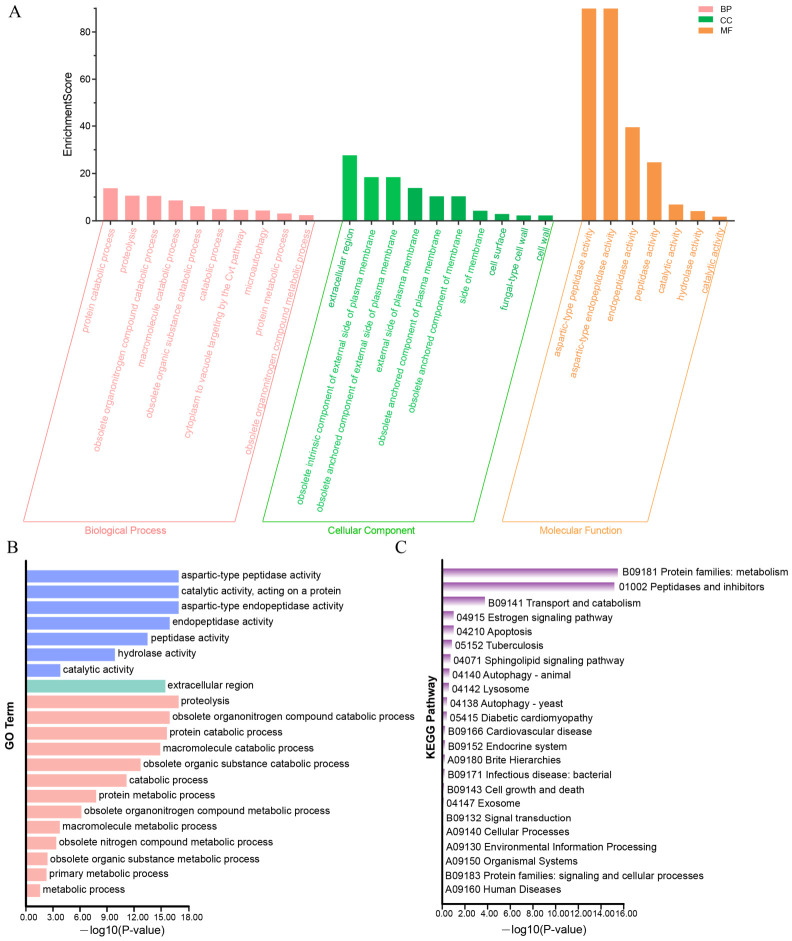
The GO annotation and KEGG pathway enrichment analysis of *TmAPs*. (**A**) The GO annotation of *TmAPs*. (**B**) The GO enrichment analysis of *TmAPs*. (**C**) The KEGG pathway enrichment analysis of *TmAPs*.

To further clarify the annotated functions of the *TmAP* genes, Gene Ontology (GO) annotation analysis was carried out based on the categories of biological process (BP), molecular function (MF), and cellular component (CC) (Figure 8A). In terms of biological processes, the majority of *TmAP* genes were found to be mainly distributed in the categories of protein catabolic process, proteolysis, obsolete organonitrogen compound catabolic process and macromolecule catabolic process, etc. Regarding molecular function, most genes were predominantly involved in aspartic-type peptidase activity, aspartic-type endopeptidase activity, endopeptidase activity and peptidase activity. And in the cellular component classification, the identified associations were found to exhibit diverse functionalities, encompassing the extracellular region, obsolete intrinsic component of external side of plasma membrane, and obsolete anchored component of external side of plasma membrane. Overall, the results of GO annotation analysis indicate that *TmAP* genes are functionally diverse and play roles in the enrichment of catabolic processes and the activation of peptidase activity.

Subsequently, GO and KEGG enrichment analyses were performed to further elucidate the function of *TmAP* genes at the molecular level. The findings of GO enrichment analysis suggested that the primary functions of these genes were involved in aspartic-type peptidase activity, catalytic activity, acting on a protein, aspartic-type endopeptidase activity, endopeptidase activity, extracellular region, proteolysis, obsolete organonitrogen compound catabolic process, protein catabolic process, and so on (Figure 8B). Notably, the significant enrichment of the GO term “extracellular region” was consistent with previous in silico subcellular localization predictions. This consistency indicates that the secretory properties of these APs were functionally related to the yeast phase of *T. marneffei*, confirming the biological significance of the predicted localization. The outcome of KEGG enrichment indicated that these APs were mainly enriched in protein families, metabolism, peptidases and inhibitors, transport and catabolism, protein families: signaling and cellular processes, the Sphingolipid signaling pathway, and others (Figure 8C). It is worth noting that multiple differentially expressed *AP* genes were significantly enriched in the apoptotic pathway, implying that their up-regulation in yeast cells may be functionally associated with the apoptotic or programmed cell death-related processes in this fungus. Collectively, these findings further confirm the associations of *TmAPs* with a variety of key biological processes, emphasizing their significant roles in catalytic activity, cellular transport, metabolic processes, and potentially cell death signaling in *T. marneffei*.

### 3.10. Gene Expression Analysis of TmAP Genes Under Various Abiotic Stresses

**Figure 9:**
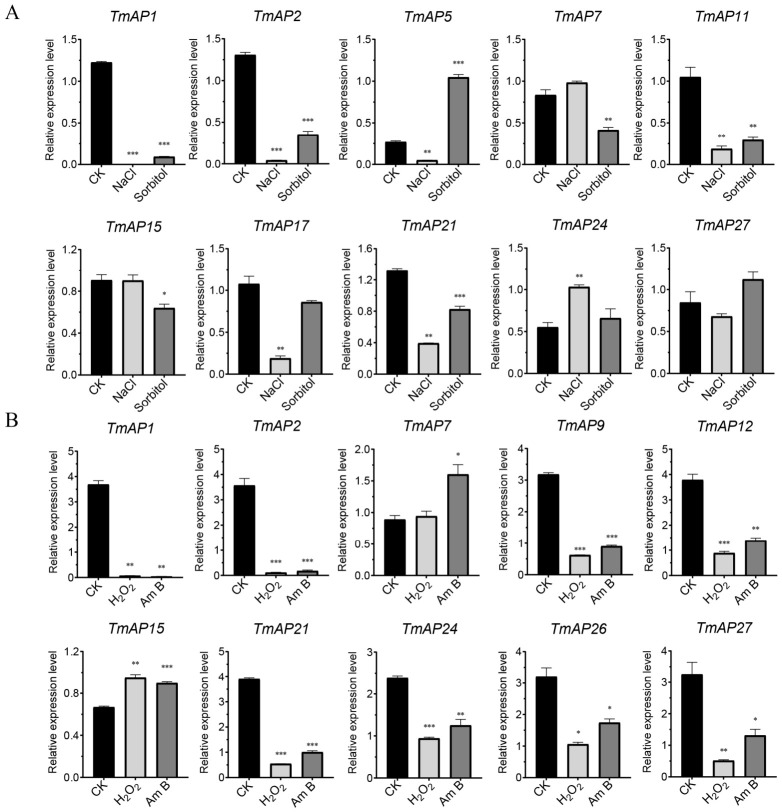
Expression analysis of *TmAP* genes in response to different stress conditions. (**A**) CK: Normal growth group; salt stress and osmotic stress simulated using NaCl (sodium chloride) and sorbitol. (**B**) Oxidative stress and antifungal stress stimulated using H_2_O_2_ (hydrogen peroxide) and amphotericin B. Statistically significant differences between CK and other treatments was determined by *t*-tests (* *p* < 0.05, ** *p* < 0.01, *** *p* < 0.001).

Once phagocytized by host cells, pathogens may encounter a range of extreme conditions, like salinity, osmosis, oxidization, and antibiotic resistance. To investigate the potential roles of the *TmAP* gene family under abiotic stress, RT-qPCR analysis was conducted using the yeast phase of *T. marneffei* to assess the expression of *TmAP* genes under salt and osmotic stress (Figure 9A). Based on the experimental results, our findings showed that the expression levels of *TmAP* genes varied significantly in response to the two distinct treatments, and most *TmAP* genes were significantly down-regulated compared with those in the normal growth group. Specifically, *TmAP5* was highly expressed under osmotic stress but showed reduced expression under salt stress, whereas *TmAP24* was highly expressed under salt stress, with no change in expression under osmotic stress.

Simultaneously, to further an additional understanding of transcript levels of *TmAP* genes in *T. marneffei* growth and development, 10 *TmAP* genes were selected and analyzed for their relative expression levels using RT-qRCR under oxidative and antifungal stress conditions (Figure 9B). Under H_2_O_2_ and amphotericin B stress, most *TmAP* genes exhibited down-regulation, except for *TmAP15* and *TmAP7*. The expression level of *TmAP15* was increased under both oxidative and antifungal stress. *TmAP7* was highly expressed under antifungal stress, but its expression did not change under oxidative stress. These *TmAP* genes with increased expression levels under various stress conditions may contribute to the resistance of *T. marneffei* to abiotic stress.

### 3.11. Protein–Protein Interaction Network and Enrichment Analysis of TmAP Proteins

**Figure 10:**
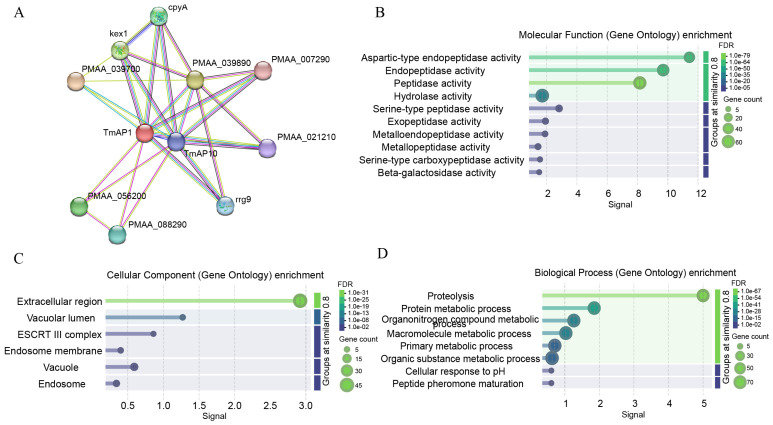
Protein–protein interaction network-based functional annotation of TmAPs. (**A**) Schematic representation of the TmAP protein–protein interaction (PPI) network; the correlation network was established using STRING analysis. Nodes indicate proteins, and lines indicate the type of interaction evidence between nodes. (**B**) Gene Ontology (GO) enrichment of *TmAPs* indicating molecular functions. (**C**) Gene Ontology (GO) enrichment of *TmAPs* indicating cellular components. (**D**) Gene Ontology (GO) enrichment of *TmAPs* indicating biological process. The sizes of the circles represent the number of genes in each category, and *x*-axis bars represent fold enrichment.

The regulatory network of protein–protein interaction (PPI) provides valuable insights into their connection functionality, which contribute to our comprehension of the gene’s function at the molecular level. To investigate the biological interactions and functional associations of TmAP proteins, a putative reciprocal network was constructed and it is shown in Figure 10A. The generated PPI network highlighted the potential roles of TmAP proteins in critical biological processes. For instance, TmAP1 and TmAP10 were predicted to interact with the pheromone-processing carboxypeptidase (Kex1), which is implicated in cell fusion and programmed cell death, indicating their potential involvement in critical cellular processes. Additionally, several TmAPs, including TmAP1 and TmAP10, were predicted to interact with Carboxypeptidase Y homolog A (CpyA), a vacuolar protease responsible for the degradation of small peptides. Moreover, TmAP1 and TmAP10 were also predicted to interact with respiratory growth protein 9 (Rrg9), a mitochondrial protein essential for respiratory activity and for the maintenance and expression of the mitochondrial genome. Collectively, these in silico predictions suggest that TmAP1 and TmAP10 are hub-like regulators with diverse functional partners. Nevertheless, experimental validation via Co-IP and yeast two-hybrid assays are urgently needed to confirm these putative interactions and to elucidate their biological relevance.

In addition, to further elucidate the annotated function of the identified TmAPs in the correlation network, we conducted functional enrichment analysis of these proteins through the online STRING tool. As depicted in Figure 10, the TmAPs were classified into three functional categories of molecular function (Figure 10B), cellular components (Figure 10C), and biological process (Figure 10D) based on Gene Ontology. The predominant biological processes mainly included proteolysis (GO: 0006508), protein metabolic process (GO: 00019538), organonitrogen compound metabolic process (GO: 1901564), macromolecule metabolic process (GO: 0043170), primary metabolic process (GO: 0044238) and organic substance metabolic process (GO: 0071704). In terms of molecular functions, the TmAPs were associated with a wide range of activities, including aspartic-type endopeptidase activity (GO: 0004190), endopeptidase activity (GO: 0004175), peptidase activity (GO: 0008233), catalytic activity acting on a protein (GO: 0140096), and serine-type peptidase activity (GO: 0008236).

### 3.12. Expression Analysis of TmAPs at Different Cultivation Conditions

**Figure 11:**
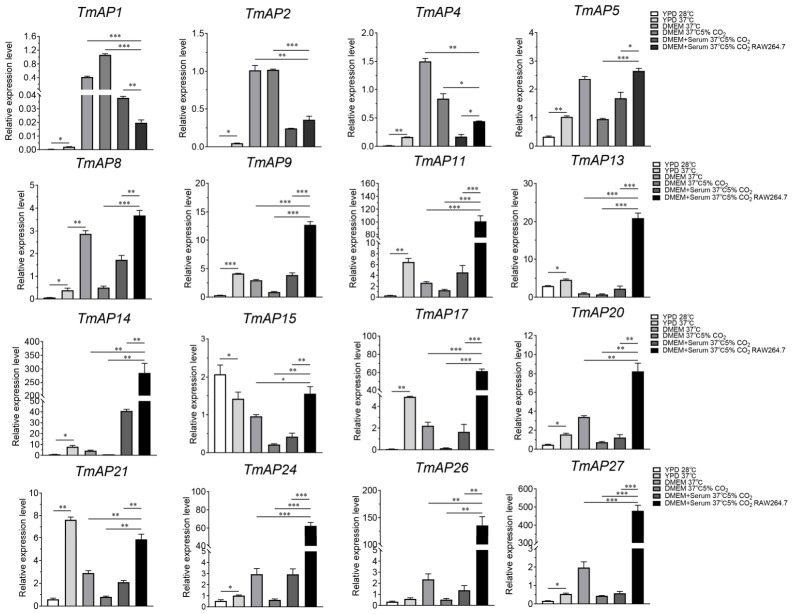
RT-qPCR analysis of *TmAP* genes in the indicated conditions. Bars represent the mean values of three technical replicates ± SE. *, **, *** indicate significant differences in *p* < 0.05, *p* < 0.01 and *p* < 0.001, respectively.

To further investigate whether the morphogenesis or culture conditions would have any impact on the expression of *TmAPs*, we conducted RT-qPCR analysis based on the *T. marneffei* incubated on either YPD (yeast extract–peptone–dextrose) or DMEM culture medium. Remarkably, as shown in Figure 11, the expression of *TmAPs* was dramatically affected by the hyphae-to-yeast transition (from YPD at 28 °C to YPD at 37 °C), with nearly all *TmAP* genes being significantly up-regulated, except for *TmAP15 and TmAP26*. These findings were consistent with the outcomes of our previous RNA-seq data.

Similarly, *TmAP* mRNA levels varied considerably when *T. marneffei* was exposed to the DMEM culture medium. Additionally, it is worth nothing that the mRNA levels of *TmAP* genes were further increased by co-culturing with murine macrophages (RAW264.7) in tissue culture medium. *T. marneffei* possesses a defining property of being able to undergo a dual-phase transformation between hyphal and yeast morphologies, with the yeast form being implicated with virulence. The above results suggest that those elevated *TmAP* genes could be vital components in growth, morphogenesis, and pathogenicity during *T. marneffei* infection.

## 4. Discussion

As a class of critical proteolytic enzymes, APs are primarily involved in protein processing and degradation and play vital roles in numerous physiological processes, such as development, reproduction, immune defense, and stress responses [45,46]. Previous studies on the *AP* gene family have mostly focused on the field of plants, whereas corresponding reports on fungi are very limited. Currently, increasing amounts of evidence have demonstrated that fungal APs are key factors contributing to the virulence and pathogenicity of disease-causing pathogens, such as the genus *Candida*, *Aspergillus*, and *Cryptococcus* [47]. Despite their extensive identification and functional roles being conducted in numerous species, systematic characterization, comprehensive information and expression analysis of the *AP* gene family in *T. marneffei* remain unexplored. The current study comprehensively identified and characterized the AP genes in *T. marneffei*, which included their molecular characterization, chromosomal distribution, phylogeny, gene structures, conserved motifs, gene duplications, and expression patterns, providing insights into their structural conservation, evolutionary relationship, and potential functional roles. Our observations provide an overall understanding of the *AP* gene family in *T. marneffei* and lay a significant foundation for further investigation of their functions in fungal growth, development, and pathogenicity.

Once internalized, invasive fungi are subjected to various extreme stress conditions within host macrophages, including temperature variation, salinity, osmosis, oxidization, and antifungal drug-induced stress. Despite encountering such diverse stress, fungal pathogens are able to replicate intracellularly, which is attributed to a large number of complex biological processes regulated by a plethora of genes. Among these, APs are well known for their significant role in the management of abiotic stress. Differential expression of APs in response to stress in many fungal species has previously been reported. Battu et al. [48] reported that aspartyl protease-dependent cleavage of the flavodoxin-like protein cgPst2 in *C. glabrata* was pivotal to the cellular oxidative stress response and survival. Another study revealed the up-regulation of seven GPI-linked aspartyl protease genes in response to acidic environmental conditions via genome-wide expression analysis [27]. Aspartyl proteases have also been reported for their roles in vacuole homoeostasis and in tolerance to high temperature and nutrient limitation. Bairwa et al. [28] uncovered novel roles of aspartyl protease in the regulation of *C. glabrata* vacuole morphogenesis by the maintenance of metal ion homoeostasis. In *Candidozyma auris*, the expression of two aspartyl protease genes was found to be increased upon exposure to nutrient starvation, NaCl, and thermal stress [49]. In the present work, four *TmAP* genes (*TmAP5*, *TmAP7*, *TmAP15*, and *TmAP24*) were significantly up-regulated under the corresponding adverse conditions, indicating their potential functions in abiotic stress responses. Although relatively few studies have been focused on the role of APs in abiotic stress tolerance in *T. marneffei*, the above observations suggest that APs may contribute to stress tolerance in this fungal pathogen.

Morphological shift is a characteristic trait of many dimorphic fungi when subjected to environmental stimuli. The ability of these pathogens to cause infection and disease is attributed to their thermo-dimorphic feature to switch from the mycelium (infective form) to yeast (pathogenic form) [50]. Recently, numerous studies have demonstrated the participation of aspartic proteases in thermal-dependent dimorphism switching. For instance, Silva et al. [22] characterized that the aspartic proteases from *Paracoccidioides brasiliensis* were modulated by fungal thermo-dimorphism, and Pepstatin A can inhibit dimorphic switching from mycelium to yeast in *P. brasiliensis*. In *Sporothrix brasiliensis*, inhibition of aspartic proteases by Pepstatin A blocked the mycelium-to-yeast dimorphic transformation, highlighting the roles of these proteases as dimorphism regulators [23]. In this study, we evaluated the expression of AP genes during the mycelium to yeast transition in *T. marneffei*. Notably, most *TmAP* genes were observed to be up-regulated during this transition, underscoring their pivotal roles in the fungal dimorphic shift to the yeast form. Similar results have been reported in other studies conducted on *Paracoccidioides lutzii* and *Paracoccidioides brasiliensis* [51,52]. In addition, through RT-qPCR and RNA-seq data analysis, it was found that the expression levels of *TmAP1* and *TmAP2* genes were most significantly increased across different phases. Further examination confirmed elevated transcriptional abundances in the yeast form compared to the mycelium form throughout all *T. marneffei* strains used in this experiment. Previous studies have postulated that, as a crucial pathogenic factor in many human fungal pathogens, aspartic proteases play significant roles in the host invasion process and morphological development. In *T. marneffei*, infection begins after inhalation of fungal spores and their subsequent thermo-dimorphic shift to the yeast form. Therefore, we speculate that *TmAP1* and *TmAP2* are modulated by environmental conditions and during fungal thermo-dimorphism and may perform key functions in the infection and pathogenecity of *T. marneffei*. However, the specific biological functions of these genes need to be further elucidated.

A typical feature of aspartic proteases is the presence of a conserved Asp domain, which enables their functions in protein processing, maturation, degradation, and signaling [11]. The active center of this domain contains two aspartic acid residues within the conserved Asp-Thr/Ser-Gly motifs and is crucial for catalytic activity [10]. In the multiple sequence alignment of APs in *T. marneffei*, all family members included a constant full-length Asp domain with a catalytic sequence, and the two aspartic residues within the active sites were highly consistent in all TmAPs, indicating their functional conservation during evolutionary processes. Similar results have also been observed in sequence comparisons of other species, suggesting that the identification results are reliable and accurate. By analyzing the conserved motif, it was found that motifs 4 and 5 contained the aspartic residue within the catalytic sequences, while motifs 6 and 7 were conserved and present in nearly all TmAP members. We speculated that these specific motifs are highly likely to be key factors in maintaining protein function. In addition, in the present study, gene structure analysis revealed that the *TmAP* genes have a distinct number of introns, with the number of introns ranging from 0 to 3. The structural variations were relatively minor, which may contribute to the functional conservation of the *TmAP* genes. Furthermore, phylogenetic analysis suggested that the gene structures of closely related members were similar. *TmAP* genes with similar motif compositions tended to cluster together, indicating that the *TmAP* genes with similar structures and evolutionary patterns probably share similar functionality. Previous studies have found that genetic variation in microbial virulence factors may shape protein functions, as well as the virulence and pathogenicity of pathogens [53,54]. Lin et al. [36] reported that a homozygous variation causing an amino acid exchange from valine to leucine, close to the proteolytic activation center of *C. albicans* Sap2, enhanced fungal pathogenicity by complement evasion and M2-like phenotype switching. Similarly, a sequence variation pattern was also observed in the homology comparison of AP proteins in *T. marneffei*, indicating the possibility of altered activity and biological function of these proteases.

As previous studies have demonstrated, gene duplication events primarily involving tandem and segmental duplications are necessary for the evolution and natural differentiation of new gene families [55,56]. To our knowledge, the expansion of the *AP* gene family through gene duplications has been extensively documented across plant species [57,58,59]. For example, tandem duplication has been observed to drive the expansion of *AP* genes in *Solanum tuberosum* [60] and *Epimedium pubescens* [61], whereas in *Populus trichocarpa* [62] and *Phyllostachys edulis* [63], the development of the *AP* gene family expansion is primarily attributed to segmental duplication. Tandem gene duplications facilitate the rapid expansion of new gene copies within genomic clusters over short evolutionary timespans, whereas segmental duplications involve long duplications of a genomic region in the entire genome with high sequence identity [64,65,66]. The expansion patterns of *AP* gene families exhibit distinguishing traits among species, which might be attributed to distinct evolutionary strategies during the adaptive process. In this study, analysis of the duplication revealed one segmental duplication event within the *TmAP* gene family. More importantly, eight repeating gene pairs duplicated in the tandem duplication regions were identified, which likely acted as a primary force to expand this gene family in *T. marneffei*. In addition, during further evolutionary selective pressure analysis, it was found that the duplicate gene pair had a *Ka/Ks* value of < 1.0, indicating that purifying selection has potentially played a dominant role in maintaining the ancestral functions of these genes by eliminating harmful mutations during evolution, thereby suggesting their critical physiological functions in *T. marneffei*.

Phylogenetic analysis revealed that the *AP* genes in *T. marneffei* are evolutionarily conserved, with the TmAP family exhibiting a closer evolutionary affinity to AP proteins from *Aspergillus* species. Further examination of gene structural features revealed that closely related TmAP members typically share comparable exon–intron arrangements, implying a common evolutionary origin. For example, six *TmAP* genes—namely, *TmAP8*, *TmAP25*, *TmAP20*, *TmAP11*, *TmAP16*, and *TmAP21*—exhibited a conserved gene architecture, with one intron and two exons at identical positions, indicting a closer evolutionary relationship among them. Similarly, another set of six *TmAP* genes (*TmAP27*, *TmAP5*, *TmAP13*, *TmAP22*, *TmAP23*, and *TmAP24*), which comprised only a single exon, clustered within a common evolutionary clade, further indicating their closer phylogenetic proximity. These findings suggest that genes sharing similar exon/intron patterns tend to possess genetic affinities and greater evolutionary conservation. Notably, TmAP3, which retained only a few conserved motifs and emerged as the basal gene within the *T. marneffei* gene family, was positioned remotely in the cross-species phylogeny. Importantly, the outlier status of TmAP3 in the broader fungal tree is not at odds with its basal position within the *T. marneffei*. The former stems from accelerated evolution and compositional divergence across species, while the latter reflects its early branching within the species-specific cluster. Hence, TmAP3 appears to have originated early but evolved independently, acquiring distinct structural features. This dual evidence underscores that ancestral duplication, coupled with differential structural evolution (e.g., intron dynamics and exon shuffling), has shaped the functional diversification of the TmAP family.

## 5. Conclusions

Aspartic proteases are important proteolytic enzymes that function in fungal growth, development, and environmental adaptation. In this study, we present an overall characterization and analysis of the *AP* genes family in the whole genome of *T. marneffei*. Through genome-wide analysis, a total of 27 *TmAP* genes were identified. Their physicochemical attributes, chromosomal distribution, gene structures, motif organization, phylogenetic association, synteny and expression profiles were comprehensively analyzed. The *AP* family genes in *T. marneffei* were identified to exhibit significant structural domains, evolutionary divergence, and varied expression profiles, implying their functional diversity. Notably, the dynamic expression of *TmAPs* under various adverse conditions highlights their potential involvement in stress responses. Furthermore, synteny and collinearity analyses revealed that tandem and segmental duplications contributed significantly to the expansion of this gene family. In summary, our findings provide a better foundation for further functional exploration of the *TmAP* gene family in growth, development, and stress adaptation, and may advance a deeper understanding of the molecular mechanisms underlying *T. marneffei* infection and pathogenicity. Nonetheless, further investigations, such as gene cloning and functional characterization, are required to substantiate the functions of these genes across various physiological and biological contexts.

## Data Availability

The original contributions presented in the study are included in the article/Supplementary Materials; further inquiries can be directed to the corresponding author.

## Supplementary Materials

The following supporting information can be downloaded at https://www.mdpi.com/article/10.3390/microorganisms14071477/s1, Table S1. Characteristics of the 18 *Talaromyces marneffei* strains included in this study; Table S2. Primers used in this study; Table S3. The nucleotide and amino acid sequences of 27 *TmAP* genes; Table S4. Physicochemical properties of TmAP proteins; Table S5. Three dimensional, secondary and transmembrane structure prediction of *TmAP*s; Table S6. The duplicated gene pairs of *TmAP* genes; Table S7. Detailed information regarding the Ka, Ks, and Ka/Ks ratio in *T. marneffei*.
