# Supplementary material for: Genome-Wide Identification and Expression Analysis of the Aspartic Protease Gene Family and Their Responses to Abiotic Stress in Talaromyces marneffei
Source: Microorganisms. 2026 Jul 6;14(7):1477. doi: 10.3390/microorganisms14071477 (PMC13414439; doi:10.3390/microorganisms14071477)
Supplement: Supplementary file 1 [file microorganisms-14-01477-s001.zip › Supplementary Table S2. Primers used in this study.pdf]

**Table S2. Primers used in this study.**

| Gene name | Primer name | Sequence(5'-3') | Length | Tm | GC% | Product length |
|---|---|---|---|---|---|---|
| *TmAP1* | F | TTTGTTGTTGACCTTCGGCG | 20 | 59.62 | 50 | 101 |
| | R | CCAGAATCGACGTATGCCCA | 20 | 59.90 | 55 | |
| *TmAP2* | F | CTCGACACCGGTAGCAACAT | 20 | 60.11 | 55 | 123 |
| | R | GGTAGCGTTGCAGGGAAAGA | 20 | 60.32 | 55 | |
| *TmAP4* | F | CCTGTGCGGCAGAATACCTT | 20 | 60.39 | 55 | 163 |
| | R | AGGAAGCATCAACCTCGACC | 20 | 59.75 | 55 | |
| *TmAP5* | F | AACCCCTGCTGTTGGATCTC | 20 | 59.67 | 55 | 132 |
| | R | ACGCGAACCTTATCCACGAA | 20 | 59.76 | 50 | |
| *TmAP6* | F | GCTCTCGACTTTCAGGCTGT | 20 | 60.04 | 55 | 125 |
| | R | GCCACAAACTCAACGTCACC | 20 | 59.97 | 55 | |
| *TmAP7* | F | GGACTGACTCTATTGCCGCT | 20 | 59.54 | 55 | 142 |
| | R | ACCCTAGGGGTTTGAGGAGT | 20 | 59.50 | 55 | |
| *TmAP8* | F | TCACGCTAGACACATGACGC | 20 | 60.46 | 55 | 102 |
| | R | GAAGCCGCCCTACCAATCAT | 20 | 60.18 | 55 | |
| *TmAP9* | F | TGGCATCGTTGGATTAGCGT | 20 | 60.11 | 50 | 128 |
| | R | GAGCTTGGTGCTTGAGGGTA | 20 | 59.68 | 55 | |
| *TmAP10* | F | AACCTCGAGGCCATCAACAG | 20 | 60.04 | 55 | 170 |
| | R | CGCTTCTGCGCAAATCCTAC | 20 | 59.97 | 55 | |
| *TmAP11* | F | TGGCAGCCTAGTTGGAGGTA | 20 | 60.25 | 55 | 148 |
| | R | GGCCACTCCCATAACACCAT | 20 | 59.74 | 55 | |
| *TmAP12* | F | CTGGCTCTGTTCGTGTCGAT | 20 | 60.11 | 55 | 157 |
| | R | TAGACGTAGTGAGTTGCGGC | 20 | 59.83 | 55 | |
| *TmAP13* | F | TGATGCCGGGTTATCACTGG | 20 | 59.82 | 55 | 154 |
| | R | TCAAGCATAGCTGAGACGGG | 20 | 59.54 | 55 | |
| *TmAP14* | F | TGGGATTTGGCTTCCTTGAGT | 21 | 59.57 | 47.62 | 128 |
| | R | GCTACCAGGGGTTAGATGCC | 20 | 59.89 | 60 | |
| *TmAP15* | F | TCAGCTTCTGGTGATGTCGG | 20 | 59.75 | 55 | 162 |
| | R | AGGCTGGACGGTATTGATGC | 20 | 60.18 | 55 | |
| *TmAP16* | F | ACGACGTTGTTATTCCGCCT | 20 | 60.04 | 50 | 181 |
| | R | TGTTTAGCAAAACCGAGCCG | 20 | 59.41 | 50 | |

| | | | | | | |
|---|---|---|---|---|---|---|
| *TmAP17* | F | ACCAACCACAGGATGGCATT | 20 | 59.89 | 50 | 112 |
| | R | AGAGGGGCATTAAGGAAGCG | 20 | 59.82 | 55 | |
| *TmAP18* | F | GTGTTGATAGCAGCCAGGGA | 20 | 59.75 | 55 | 184 |
| | R | TTCGCGGAGTCGTAATAGGC | 20 | 59.97 | 55 | |
| *TmAP20* | F | TTCAGATCGGCCAGGTTACG | 20 | 59.83 | 55 | 110 |
| | R | GGCCAATCCCACGATACCAT | 20 | 59.85 | 55 | |
| *TmAP21* | F | TGGGTCTTTGATTGCAGGGA | 20 | 59.22 | 50 | 130 |
| | R | GCTGGATACCGCCATAGCAA | 20 | 60.25 | 55 | |
| *TmAP24* | F | CCCCTGGTATTGGAACCCAC | 20 | 60.03 | 60 | 121 |
| | R | TTGTCCTGGAACACACGACC | 20 | 60.18 | 55 | |
| *TmAP26* | F | CCGATAGGGGCTTATGGCAG | 20 | 60.04 | 60 | 101 |
| | R | AGCAGAGTCCAGAACAGCAC | 20 | 59.97 | 55 | |
| *TmAP27* | F | CACACAGGCGCTCAATCCTA | 20 | 60.11 | 55 | 170 |
| | R | ACATACCAGGTGCCTCATGC | 20 | 60.11 | 55 | |
| *β-Actin* | F | TGATGAGGCACAGTCTAAGC | 20 | 57.32 | 50 | 190 |
| | R | CTTCTCTCTGTTGGACTTGG | 20 | 55.40 | 50 | |
