# Supplementary material for: Genome-Wide Identification and Expression Analysis of the Aspartic Protease Gene Family and Their Responses to Abiotic Stress in Talaromyces marneffei
Source: Microorganisms. 2026 Jul 6;14(7):1477. doi: 10.3390/microorganisms14071477 (PMC13414439; doi:10.3390/microorganisms14071477)
Supplement: Supplementary file 1 [file microorganisms-14-01477-s001.zip › Supplementary Table S5. Three dimensional, secondary and transmembrane structure prediction of TmAPs.pdf]

Table S5. Three dimensional, secondary and transmembrane structure prediction of TmAPs.

Gene names	3D structure	Secondary structure	Transmembrane helices
TmAP1	Sequence identity: 99.49% GMQE: 0.88 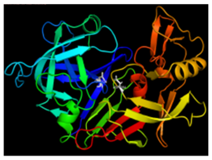	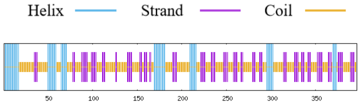	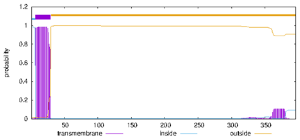
TmAP2	Sequence identity: 96.82% GMQE: 0.86 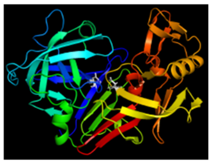	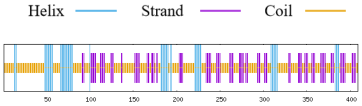	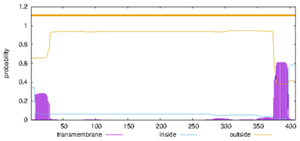
TmAP3	Sequence identity: 96.20% GMQE: 0.86 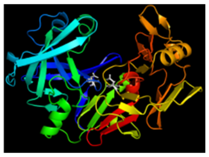	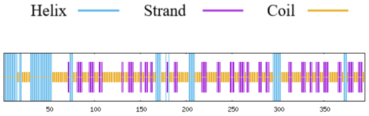	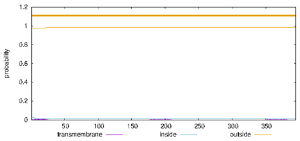
TmAP4	Sequence identity:99.75% GMQE: 0.87 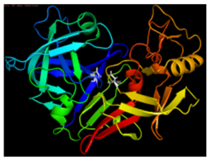	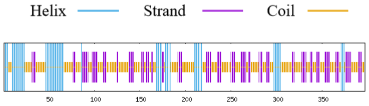	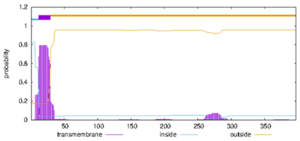
TmAP5	Sequence identity:99.49% GMQE: 0.88 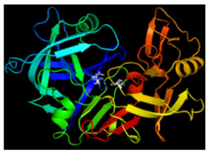	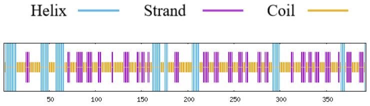	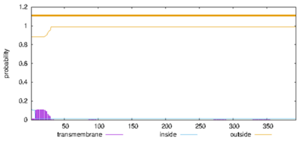
TmAP6	Sequence identity:100% GMQE: 0.84 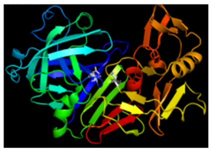	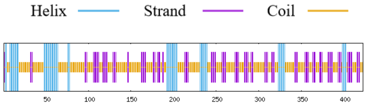	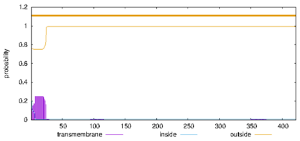
TmAP7	Sequence identity:100% GMQE: 0.87 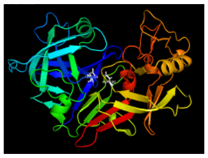	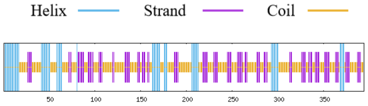	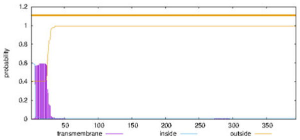

TmAP8

Sequence identity:98.98%
GMQE: 0.86

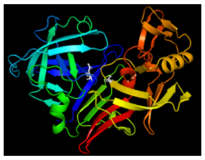

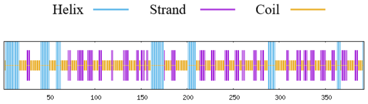

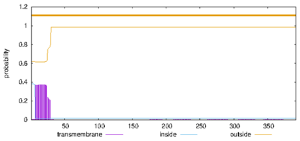

TmAP9

Sequence identity:99.50%
GMQE: 0.88

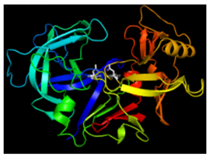

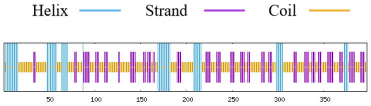

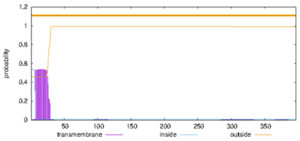

TmAP10

Sequence identity:100%
GMQE: 0.84

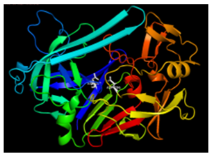

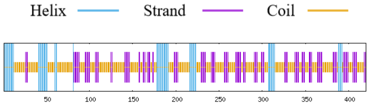

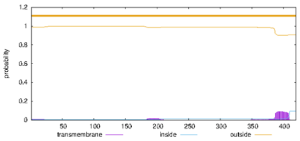

TmAP11

Sequence identity:99.24%
GMQE: 0.87

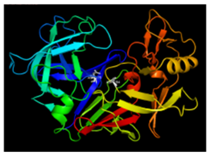

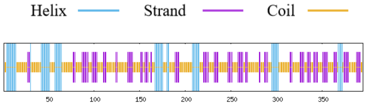

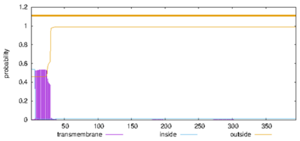

TmAP12

Sequence identity:99.75%
GMQE: 0.80

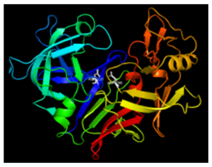

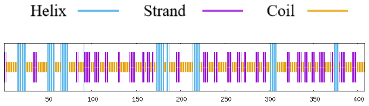

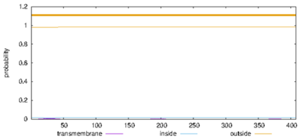

TmAP13

Sequence identity:99.75%
GMQE: 0.89

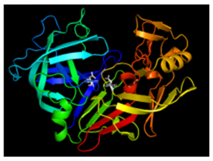

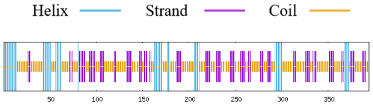

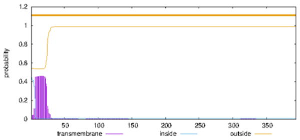

TmAP14

Sequence identity:98.99%
GMQE: 0.86

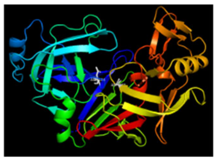

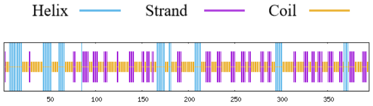

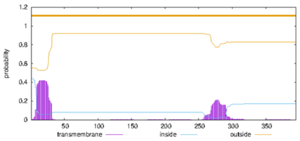

TmAP15

Sequence identity:75.76%
GMQE: 0.89

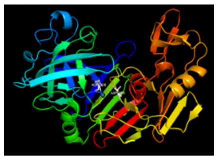

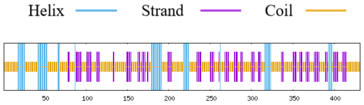

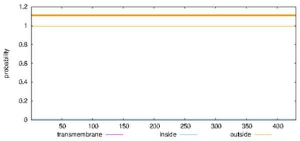

Sequence identity:99.49%
GMQE: 0.84

TmAP16

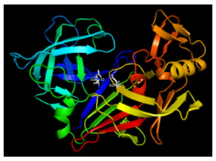
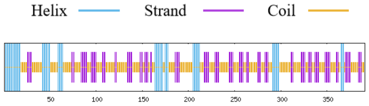

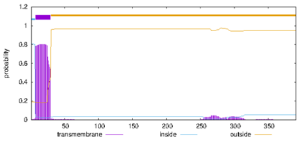

Sequence identity:80.96%
GMQE: 0.87

TmAP17

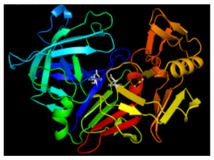
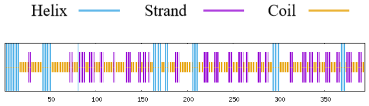

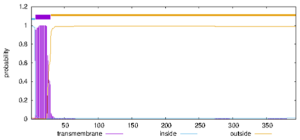

Sequence identity:99.49%
GMQE: 0.88

TmAP18

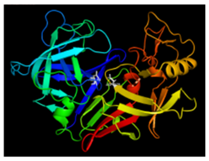
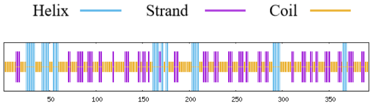

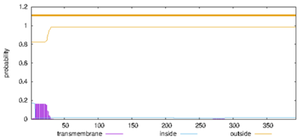

Sequence identity:99.75%
GMQE: 0.87

TmAP19

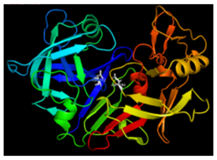
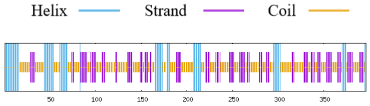

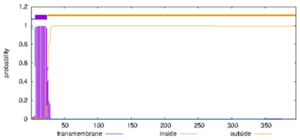

Sequence identity:100%
GMQE: 0.84

TmAP20

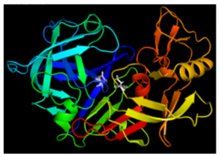
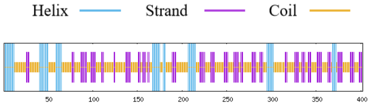

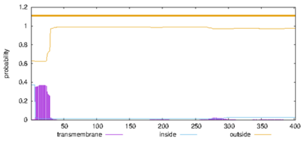

Sequence identity:100%
GMQE: 0.86

TmAP21

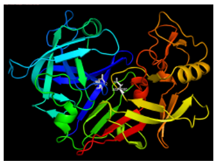
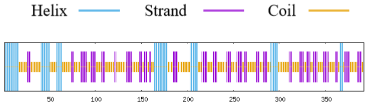

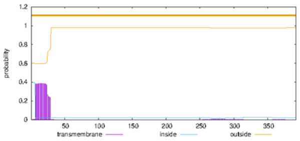

Sequence identity:99.49%
GMQE: 0.89

TmAP22

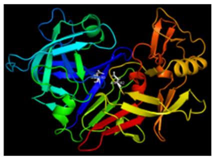
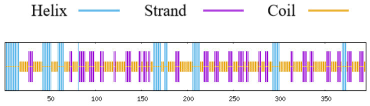

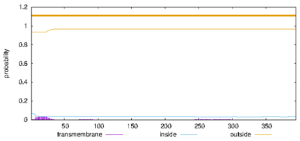

Sequence identity:98.98%
GMQE: 0.88

TmAP23

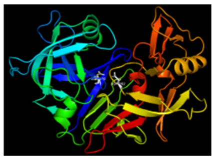
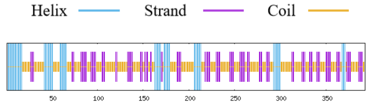

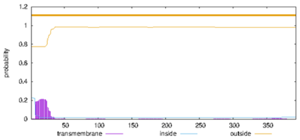

TmAP24	Sequence identity:100%<br>GMQE: 0.88<br>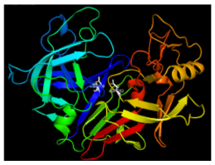	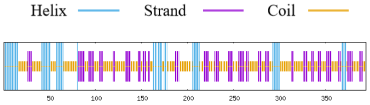	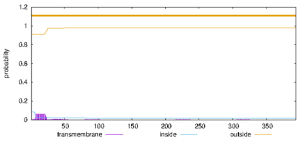
TmAP25	Sequence identity:98.99%<br>GMQE: 0.86<br>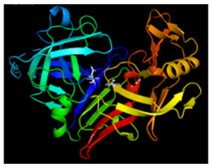	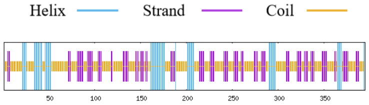	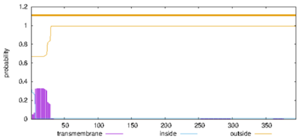
TmAP26	Sequence identity:99.21%<br>GMQE: 0.85<br>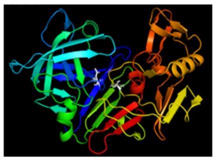	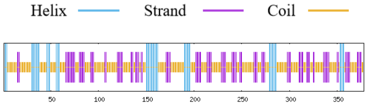	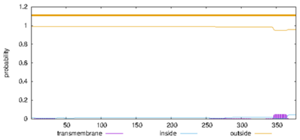
TmAP27	Sequence identity:99.20%<br>GMQE: 0.88<br>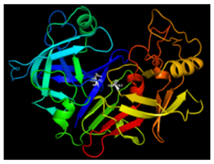	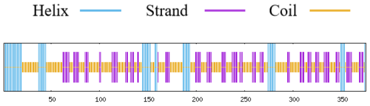	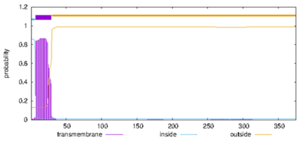
